# Cost-Effectiveness of Periodontal Intervention Combined with Diabetes Management in China: A Markov Analysis

**DOI:** 10.1016/j.identj.2026.109750

**Published:** 2026-07-18

**Authors:** Xinhai Yin, Yuhan Dai, Guohui Bai, Jukun Song

**Affiliations:** a Department of Oral and Maxillofacial Surgery, Guizhou Provincial People’s Hospital, Guiyang, China; b The Affiliated Stomatological Hospital of Zunyi Medical University, Zunyi, China; c Department of Oral and Maxillofacial Surgery, the Affiliated Stomatological Hospital of Guizhou Medical University, Guiyang, China

**Keywords:** Periodontitis, Type 2 diabetes mellitus, Cost-utility analysis, Markov model, China, Quality-adjusted life years, Integrated management

## Abstract

**Background:**

To evaluate the long-term cost-effectiveness of integrating periodontal intervention with diabetes management for Chinese adults with comorbid type 2 diabetes mellitus (T2DM) and periodontitis.

**Methods:**

A 9-state Markov cohort model (1-year cycle; 40-year horizon) was constructed for a hypothetical cohort of 50-year-old Chinese adults with comorbid T2DM and periodontitis. Three strategies were compared: usual care (UC), scaling and root planing plus intensified diabetes management (SRP+DM), and comprehensive integrated management (CIM). Transition probabilities, costs (2024 CNY; 5% discount rate), and utilities were derived from Chinese national data and systematic reviews. One-way and probabilistic sensitivity analyses (PSA; 10,000 Monte Carlo simulations) were performed. Willingness-to-pay (WTP) thresholds were CNY 90,000/QALY (1 × GDP per capita) and CNY 270,000/QALY.

**Results:**

Usual care generated CNY 104,028 and 11.749 QALYs. SRP+DM yielded an incremental cost-utility ratio (ICUR) of CNY 5,308/QALY versus UC; CIM yielded CNY 6,608/QALY. Both strategies were cost-effective at CNY 90,000/QALY. In patients with uncontrolled diabetes (HbA1c ≥7%), both were cost-saving. CIM reduced cumulative 40-year DM complication incidence by 11.9 percentage points, tooth loss by 12.7 percentage points, and all-cause mortality by 4.8 percentage points versus UC. CIM was cost-optimal in 99.4% of simulations.

**Conclusions:**

Subject to the model's assumptions—notably extrapolation of short-term randomized-trial effects over a lifetime, pooled estimates of periodontal recurrence and HbA1c-benefit durability, and indirectly derived utilities—integrated periodontal–diabetes management appears cost-effective in China across all WTP thresholds tested in deterministic and probabilistic sensitivity analyses. In durability scenario analyses, this conclusion remained robust when the treatment effect was lost after 1, 3, or 5 years, except when the benefit disappeared within 1–3 years while maintenance costs continued. These findings support including periodontal intervention in China's national diabetes care pathway but require confirmation by long-term empirical studies.

## Introduction

The global burden of type 2 diabetes mellitus (T2DM) and periodontitis continues to escalate. China bears the world's largest absolute burden of T2DM, with an estimated 140 million adults affected as of 2021, representing approximately 12.8% of the adult population.^1^ The 4th National Oral Health Survey (2015-2016) reported that the prevalence of moderate-to-severe periodontitis exceeded 52% in Chinese adults aged 35 to 44 years, rising to above 70% in those aged 55 to 74 years.^2^^,^^3^ The coexistence of T2DM and periodontitis is clinically important: hyperglycaemia impairs neutrophil function and promotes an exaggerated inflammatory response that accelerates alveolar bone destruction, whilst the systemic inflammatory burden of periodontitis worsens insulin resistance and glycaemic control.^4^ Throughout this analysis, ‘non-surgical periodontal therapy’ is defined according to the European Federation of Periodontology (EFP) S3 clinical practice guideline as a structured 3-step protocol comprising (i) supragingival biofilm and risk-factor control with oral hygiene instruction; (ii) subgingival instrumentation by full-mouth scaling and root planing (SRP); and (iii) periodontal supportive therapy (SPT)—recall maintenance visits at individualised intervals (typically 3–6 months) for re-evaluation, professional biofilm removal, and re-instrumentation of residual pockets—which is recognised as an essential and ongoing component of effective non-surgical periodontal care rather than an optional adjunct.

A landmark Cochrane systematic review and meta-analysis by Simpson et al.,^5^ which included 35 randomised controlled trials enrolling 3,249 adults with type 1 or type 2 diabetes mellitus and periodontitis, demonstrated that non-surgical periodontal therapy—chiefly subgingival scaling and root planing (SRP) supplemented by oral hygiene instruction and professional mechanical plaque removal—reduced glycated haemoglobin (HbA1c) by a mean of 0.43% (95% confidence interval [CI]: −0.59% to −0.28%; 30 studies, 2,443 participants) at 3–4 months post-intervention; pooled estimates also showed reductions of 0.30% (95% CI: −0.52% to −0.08%; 12 studies, 1,457 participants) at 6 months and 0.50% (95% CI: −0.55% to −0.45%; 1 study, 264 participants) at 12 months.^5^ The 2022 update doubled the number of included studies relative to the 2015 version and provides moderate-certainty evidence that periodontal treatment improves glycaemic control by a clinically significant amount; however, sex- and age-stratified subgroup data were not separately reported in the published meta-analysis. Comprehensive integrated management (CIM) programmes that combine SRP and supportive periodontal therapy with intensified glycaemic monitoring, individualised dietary counselling, and structured patient education have reported larger HbA1c reductions: a 2025 systematic review and meta-analysis by Umezaki et al. (11 RCTs) reported pooled HbA1c reductions of 0.64% (95% CI: −0.96% to −0.32%; I²=73%) at 3 months and 0.33% (95% CI: −0.65% to −0.01%) at 6 months.^6^ Each 1% reduction in HbA1c is associated with approximately 21% fewer diabetes-related endpoints and 37% fewer microvascular complications.^7^

Despite compelling clinical evidence, the economic value of incorporating periodontal treatment into diabetes care in China has not been rigorously assessed. Several cost-effectiveness analyses from high-income settings report favourable incremental cost-utility ratios (ICURs),^8^, ^9^, ^10^ but their applicability to China is limited by differences in treatment costs, disease epidemiology, healthcare system structure, and willingness-to-pay (WTP) benchmarks.^11^ Compared with these prior analyses—most of which adopted simpler decision-tree or 3-state Markov structures, modelled only 1 or 2 periodontitis severity strata, and did not stratify by glycaemic control—the present study aims to provide a more granular, China-specific economic evaluation by (i) explicitly modelling 6 joint DM–periodontitis health states stratified by both glycaemic control (HbA1c <7% vs ≥7%) and periodontitis severity (healthy/mild–moderate/severe per the 2018 EFP/AAP staging classification); (ii) populating all transition probabilities, costs, and utilities exclusively from Chinese national or contemporary Chinese published sources; (iii) directly comparing 3 contemporary management strategies—usual care (UC), SRP plus intensified diabetes management (SRP+DM), and comprehensive integrated management (CIM); and (iv) performing pre-specified subgroup analyses by baseline glycaemic control to inform targeted policy recommendations. We constructed a Markov cohort model to evaluate the long-term clinical and economic outcomes of these 3 strategies for Chinese adults with comorbid T2DM and periodontitis.

## Methods

### Analytic model

A Markov cohort model was developed with a 1-year cycle length and a 40-year (lifetime) horizon to simulate outcomes for a hypothetical cohort of 50-year-old Chinese adults with comorbid T2DM and periodontitis, consistent with the peak prevalence of this comorbidity in China.^2^^,^^12^ The model was designed and reported in accordance with CHEERS 2022.^13^

**Fig. 1:**
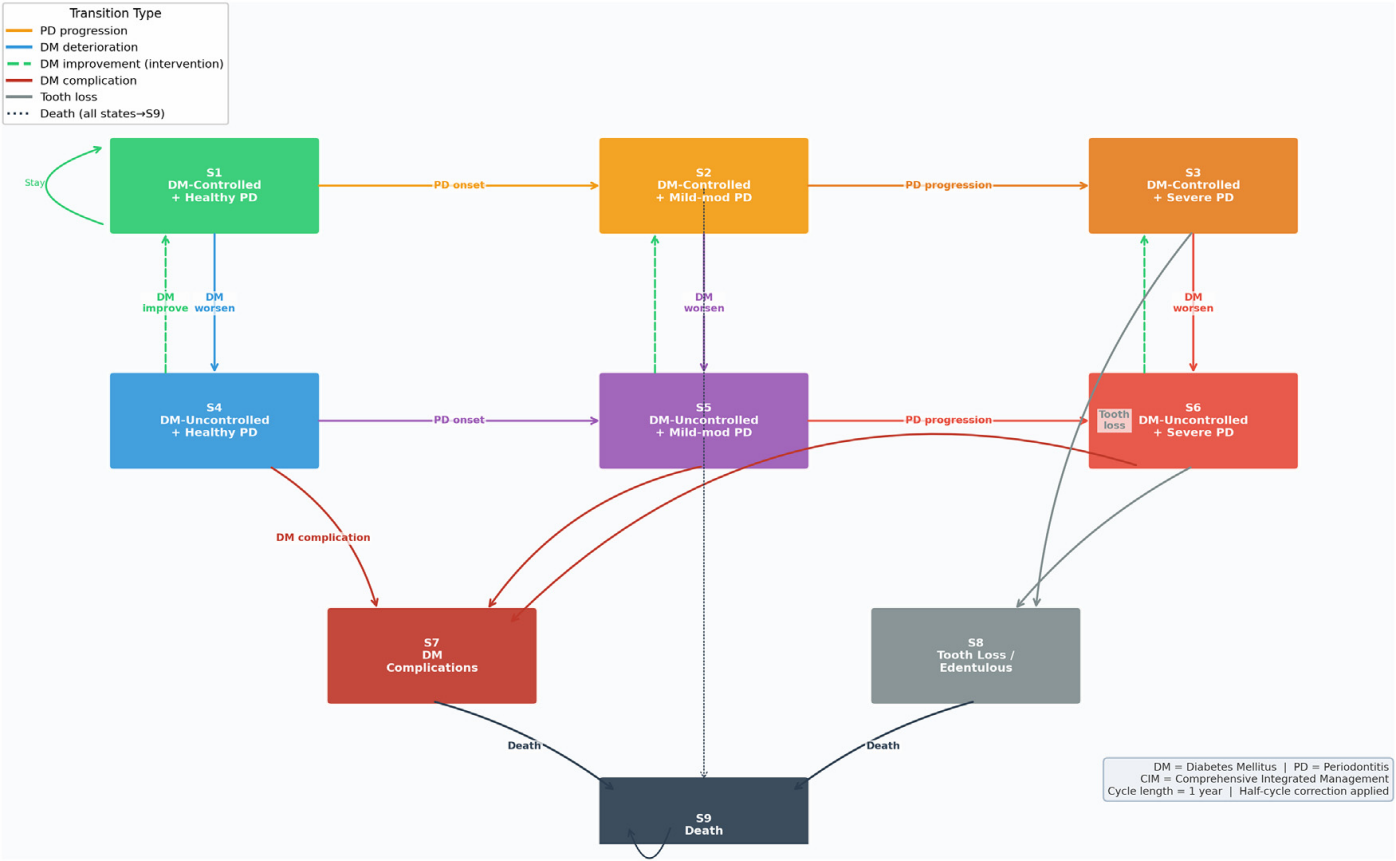
Markov state-transition diagram. Health states: S1, DM-controlled with healthy periodontium; S2, DM-controlled with mild–moderate PD; S3, DM-controlled with severe PD; S4-S6, the corresponding DM-uncontrolled states; S7, DM complications; S8, tooth loss/edentulous; S9, death (absorbing). Arrow colours denote transition type: orange, PD progression; blue, DM deterioration; green dashed, intervention-enabled DM improvement; red, complication onset; grey, tooth loss; dotted, background mortality from all living states. DM, diabetes mellitus; PD, periodontitis.

The model incorporated 9 mutually exclusive health states: (S1) controlled DM with healthy periodontium; (S2) controlled DM with mild-to-moderate periodontitis; (S3) controlled DM with severe periodontitis; (S4) uncontrolled DM with healthy periodontium; (S5) uncontrolled DM with mild-to-moderate periodontitis; (S6) uncontrolled DM with severe periodontitis; (S7) DM complications (nephropathy, retinopathy, or cardiovascular events); (S8) tooth loss/edentulous state; and (S9) death (absorbing state). Allowable transitions are illustrated in Figure 1. A half-cycle correction was applied throughout.

Glycaemic-control status was defined a priori following the 2024 Chinese Diabetes Society (CDS) Guideline^14^ and the American Diabetes Association Standards of Care: ‘DM-controlled’ denoted HbA1c <7.0% (53 mmol/mol) maintained over the most recent 2 consecutive measurements at intervals of ≥3 months, and ‘DM-uncontrolled’ denoted HbA1c ≥7.0% on the same criterion. We adopted a binary classification (rather than finer HbA1c strata) for 3 reasons: (i) the 7.0% threshold is the operative target endorsed by both the CDS Guideline and major international guidelines and is the level at which Chinese reimbursement and management pathways shift; (ii) all source studies for the transition probabilities and HbA1c-modifying treatment effects (Simpson et al.^5^; Umezaki et al.^6^; Quan et al. CHIME^11^) reported outcomes against this same dichotomy, allowing internally consistent parameterisation; and (iii) finer stratification (e.g., 7%-8% vs >8%) would have introduced cells with sparse Chinese-specific evidence and increased structural uncertainty without improving face validity. Periodontitis status and severity were operationalised in line with the 2018 EFP/AAP World Workshop classification^2^^,^^3^: ‘healthy/no periodontitis’ corresponded to the absence of clinical attachment loss (CAL) attributable to periodontitis with bleeding on probing <10%; ‘mild-to-moderate periodontitis’ corresponded to Stage I-II disease (interdental CAL 1-4 mm, probing depth ≤5 mm, horizontal bone loss limited to the coronal third); and ‘severe periodontitis’ corresponded to Stage III-IV disease (interdental CAL ≥5 mm, probing depth ≥6 mm, vertical bone loss ≥3 mm and/or tooth loss attributable to periodontitis). Disease grade (rate of progression) was incorporated probabilistically through age- and HbA1c-conditioned transition rates between mild–moderate and severe states (Grade B/C progression,^2^^,^^12^), and disease extent was operationalised at the patient level by treating ‘severe periodontitis’ as generalised (>30% of teeth affected), consistent with the dominant phenotype reported in the 4th Chinese National Oral Health Survey.^2^

Three competing strategies were compared: (1) *Usual Care (UC):* standard diabetes management without active periodontal treatment; (2) *SRP+DM:* standard diabetes management plus full-mouth subgingival SRP followed by periodontal maintenance; (3) *Comprehensive Integrated Management (CIM):* all SRP+DM components plus structured patient education, dietary counselling, and intensified glycaemic monitoring, consistent with the Nanjing RCT regimen.^15^

In line with the EFP S3 guideline, both active strategies (SRP+DM and CIM) explicitly modelled periodontal supportive therapy (SPT) as an integral component of non-surgical periodontal treatment. The SRP+DM arm was operationalised as one course of full-mouth subgingival SRP at baseline followed by SPT recall visits at 3-month intervals during the first year (i.e., 4 SPT visits per patient in year 1) and 6-month intervals thereafter (2 SPT visits per patient per year from year 2 onward), comprising re-assessment, supragingival/subgingival professional biofilm removal, and re-instrumentation of any residual or recurrent pockets. The CIM arm followed the same SPT schedule but additionally included quarterly diabetes–periodontal co-management contacts and an annual structured patient-education and dietary-counselling session. The unit cost of each SPT visit (CNY 280; Table 2) and the annual frequency of SPT visits were both varied in deterministic and probabilistic sensitivity analyses to account for plausible variation in real-world adherence to recall.

### Parameters

Transition probabilities and treatment-effect parameters are presented in Table 1. Natural history transition probabilities for DM–periodontitis comorbidity were derived from Chinese national data: annual rates of periodontitis progression and tooth loss from the 4th National Oral Health Survey^2^; DM deterioration rates from Wang et al.^1^ and the Chinese Diabetes Society Guidelines 2024^14^; DM complication probabilities from the CHIME simulation model validated for Chinese T2DM patients,^11^ supplemented by national epidemiological data^16^; and age-specific background mortality from the China Health Statistics Yearbook 2022^17^ and WHO Global Health Observatory life tables. Treatment-related transition modifiers were based on meta-analytic evidence: the HbA1c-reducing effect of SRP (−0.43% at 3–4 months) from the Cochrane review by Simpson et al.^5^; the additional benefit of CIM (−0.64% at 3 months) from Umezaki et al.^6^ Mortality was modelled as 2 explicit components. Age-specific background all-cause mortality d(age), calibrated to Chinese life tables^17^ with a Gompertz form, was applied to every living state. Disease-related excess mortality was then added by one of 2 mechanisms according to state: for the established diabetic-complication state (S7) the excess hazard was additive (annual death probability = complication-specific hazard + d(age)), reflecting an event-driven risk that is largely independent of the background age gradient; whereas for the poor-glycaemic-control states (S4–S6) and the edentulous state (S8) the excess risk was multiplicative (a standardised-mortality-ratio applied to d(age): × 1.5 for S4–S5, × 1.8 for S6, and × 1.2 for S8). All death probabilities were capped at 1.0.

**Table 1:** Base-case model parameters: transition probabilities (Panel A) and treatment-effect estimates (Panel B) for usual care (UC), SRP+DM, and comprehensive integrated management (CIM).

Initial State / Parameter	Transition / Outcome	UC base % (range)	SRP+DM modifier	CIM modifier	Key Reference
***A. Natural History Transition Probabilities (annual %)***					
S1→S2: DM-ctrl + Healthy PD → Mild-mod PD	PD onset	4.5 (3.0–6.5)	RR 0.63 (0.48–0.80)	RR 0.55 (0.40–0.72)	Jiao et al., J Clin Periodontol 2021
S1→S4: DM-ctrl + Healthy → DM-uncontrolled + Healthy	DM deterioration	8.0 (5.5–11.0)	Achievement rate: 25%/yr (18–33)	Achievement rate: 32%/yr (25–39)	Wang et al., JAMA 2021; CDS Guideline 2024
S2→S3: DM-ctrl + Mild-mod PD → Severe PD	PD progression	8.0 (5.5–11.0)	RR 0.63	RR 0.55	Jiao et al. 2021; Simpson et al., Cochrane 2022
S2→S8: Mild-mod PD → Tooth loss	Tooth loss	1.2 (0.6–2.0)	RR 0.58 (0.41–0.80)	RR 0.48 (0.33–0.68)	Jiao et al. 2021; Periodontal RCT meta-analysis
S5→S7: DM-uncontrolled + Mild-mod PD → DM complications	DM complication	3.5 (2.0–5.5)	RR 0.89 (0.79–1.00)	RR 0.82 (0.71–0.94)	Quan et al., PLoS Med 2021 (CHIME); Xu et al., Lancet PH 2024
S6→S7: DM-uncontrolled + Severe PD → DM complications	DM complication	6.8 (4.5–9.5)	RR 0.82	RR 0.82	Quan et al. 2021; Xu et al. 2024
S3→S6: DM-ctrl + Severe PD → DM-uncontrolled + Severe PD	DM deterioration	5.0 (3.0–7.5)	Achievement rate modifier	Achievement rate modifier	Quan et al. 2021; CDS Guideline 2024
S7→Death (S9)	Death (complication)	5.5 (3.5–8.0)	—	—	NHC Yearbook 2022; WHO Life Tables 2021
S8→Death (S9)	Death (tooth loss)	3.0 (1.8–4.5)	—	—	NHC Yearbook 2022; WHO Life Tables 2021
***B. Treatment-Effect Parameters***					
HbA1c reduction (%, annual)	Glycaemic improvement	0 (reference)	−0.43 (−0.59 to −0.28)	−0.64 (−0.96 to −0.32)	Simpson et al., Cochrane 2022; Umezaki et al. 2025
Durability of glycaemic improvement (%/yr)	Persistence of benefit	—	75 (60–90)	80 (65–90)	Model assumption (extrapolated from Zhang et al., Sci Rep 2025; see Methods)
PD recurrence after SRP (%/yr)	Periodontal recurrence	—	12.0 (8.0–18.0)	8.0 (5.0–13.0)	Model assumption (annualised from cumulative data in Jiao et al. 2017 and Leow et al. 2022; see Methods)

Note: Annual transition probabilities (%) for UC; modifiers (relative risk or absolute rate) applied for SRP+DM and CIM. Background all-cause mortality is applied additively via age-specific life tables (WHO Global Health Estimates 2021; NHC China Health Statistics Yearbook 2022). Ranges represent 95% CI or plausible bounds used in one-way sensitivity analyses. The annual durability of glycaemic improvement (75%/yr for SRP+DM, 80%/yr for CIM) and the annual periodontitis recurrence rates (12%/yr for SRP+DM, 8%/yr for CIM) are model assumptions: source RCTs and systematic reviews report only short-term (3–12 months) HbA1c persistence and 5–20 year cumulative recurrence/tooth-loss prevalence rather than direct annual rates, so we annualised these data using a constant-rate exponential assumption. Both sets of parameters were varied across the wide plausible ranges shown to test robustness, and full derivation steps are provided in the Supplementary Material (Parameter Source Detail). Abbreviations: CDS, Chinese Diabetes Society; CHIME, Chinese Hong Kong Integrated Modelling and Evaluation; CIM, comprehensive integrated management; DM, diabetes mellitus; PD, periodontitis; RR, relative risk; SRP, scaling and root planing; UC, usual care.

Two model parameters required additional derivation because no published source reports them as direct annual rates over a lifetime horizon. *First, the annual durability of glycaemic improvement* (Table 1, Panel B; 75%/yr for SRP+DM and 80%/yr for CIM) was derived from the 3- to 6-month between-group HbA1c differences observed in the Nanjing community-based RCT by Zhang et al.^15^ and the 3-, 6-, and 12-month pooled HbA1c reductions reported in Simpson et al. and Umezaki et al.,^5^^,^^6^ using a constant-rate exponential decay model fitted to the trial-level point estimates and assuming continued SPT recall (Table 2). We emphasise that no source RCT directly reports a year-on-year persistence rate; the values used are model assumptions calibrated to the available short-term trial data and are varied widely (60%-90%/yr) in deterministic and probabilistic sensitivity analyses. *Second, the annual periodontitis recurrence rates* (12%/yr for SRP+DM, 8%/yr for CIM) were similarly derived rather than directly extracted: Leow et al.^17^ reported 5- to 20-year cumulative prevalence of tooth loss (9.6%, 95% CI 5%-14%) and CAL loss ≥2 mm at one or more sites (24.8%, 95% CI 11%-38%) under structured supportive periodontal care, and Jiao et al.^16^ reported short-term residual pocket and CAL outcomes in a large Chinese NSPT cohort. Annual recurrence rates were derived from the Leow et al. cumulative figures using a constant-rate exponential transformation across the midpoint of the 5-20-year reporting window, then adjusted upward for the SRP+DM arm to reflect lower SPT-adherence in routine community care, and adjusted downward for the CIM arm to reflect more intensive periodontal–diabetes co-management; the resulting parameters were varied across the plausible ranges shown in Table 1 (5%-18%/yr) in sensitivity analyses. A full step-by-step derivation of all model parameters, including the source values, transformation equations, and rationale for plausible ranges, is provided as Supplementary Material.

**Table 2:** Base-case cost estimates (2024 CNY) and health state utility values used in the Markov model.

Parameter	Mean (CNY or utility)	Range (SA)	Distribution	Reference
***Intervention Costs (CNY per patient/year)***				
Usual Care (DM management)	4,320	3,200–5,800	Gamma	NHSA schedule 2024 (Beijing/Shanghai)
Subgingival SRP, full mouth (per course)	1,850	1,400–2,500	Gamma	Public hospital fee schedule
Periodontal maintenance (per visit)	280	200–380	Gamma	Outpatient fee schedule
CIM patient education programme (per year)	420	300–600	Gamma	Community health centre rate
Oral hypoglycaemic agents (per year)	2,400	1,800–3,200	Gamma	NHSA Reimbursement Drug List 2024 (医保发[2024]33号)
Insulin therapy (per year)	5,800	4,500–8,000	Gamma	NHSA Reimbursement Drug List 2024
***Complication & Disease Costs (CNY per patient/year unless noted)***				
Diabetic nephropathy management	28,500	22,000–38,000	Gamma	NHSA DRG/DIP 2.0 2024; Liu et al. Front Public Health 2023
Diabetic retinopathy treatment	18,200	14,000–25,000	Gamma	NHSA DRG/DIP 2.0 2024; Huang et al. PLoS One 2016
Cardiovascular event (per event)	42,000	32,000–56,000	Gamma	NHSA DRG/DIP 2.0 2024; Liu et al. 2023
Full denture restoration (lifetime)	15,600	10,000–22,000	Gamma	Beijing/Shanghai MHSB prosthetics schedules 2024
***Health State Utilities (EQ-5D-5L Chinese value set)***				
S1: DM-controlled + Healthy PD	0.847	0.785–0.909	Beta	Luo et al., Value Health 2017
S2: DM-controlled + Mild-moderate PD	0.812	0.670–0.954	Beta	Luo et al. 2017
S3: DM-controlled + Severe PD	0.768	0.600–0.936	Beta	Luo et al. 2017
S4: DM-uncontrolled + Healthy PD	0.798	0.660–0.936	Beta	Luo et al. 2017; Zhang et al., Diabetes Ther 2020
S5: DM-uncontrolled + Mild-moderate PD	0.749	0.591–0.907	Beta	Luo et al. 2017; Zhang et al. 2020
S6: DM-uncontrolled + Severe PD	0.694	0.512–0.876	Beta	Huang et al., Front Med 2025; Zhang et al. 2020
S7: DM complications	0.631	0.435–0.827	Beta	Huang et al. 2025; Zhang et al. 2020
S8: Tooth loss / Edentulous	0.712	0.548–0.876	Beta	He & Wang, Qual Life Res 2015
S9: Death	0.000	—	Fixed	—

Note: All costs in 2024 Chinese Yuan (CNY). Costs and QALYs discounted at 5%/year. Ranges represent plausible bounds for sensitivity analyses. Gamma distributions assigned to cost parameters; Beta distributions to utility values. Cost sources: Beijing/Shanghai Municipal Healthcare Security Bureau fee schedules (2024); NHSA National Reimbursement Drug List 2024 (医保发〔2024〕33号, effective 1 January 2025); NHSA DRG/DIP 2.0 national inpatient grouping framework (July 2024). Health-state utilities for composite DM–PD states (S1–S6) were derived by anchoring base utilities to published Chinese EQ-5D-3L/5L observations of the corresponding DM-control category^18^^,^^19^ and applying an additive periodontitis-severity disutility increment (approximately −0.035 for mild–moderate and −0.079 for severe disease) estimated from OHIP-14 score differences reported in Jiao et al. and the 4th National Oral Health Survey^2^^,^^12^ using a published OHIP-to-EQ-5D mapping algorithm. The utility for the edentulous state (S8) is similarly an indirect derivation, transformed from the OHIP-EDENT-C score range reported by He & Wang (2015)^20^ in Chinese complete-denture wearers, rather than a directly elicited EQ-5D utility. Both the indirect mapping and absolute utility ranges are varied widely in sensitivity analyses to capture the resulting structural and parametric uncertainty. A full step-by-step derivation, including source-value tables and transformation equations, is provided as Supplementary Material (Table S1: Parameter Source Detail). Abbreviations: CIM, comprehensive integrated management; CNY, Chinese Yuan; EQ-5D-3L/5L, EuroQol 5-dimension 3-level/5-level; MHSB, Municipal Healthcare Security Bureau; NHSA, National Healthcare Security Administration; OHIP, Oral Health Impact Profile; PD, periodontitis; SA, sensitivity analysis; SPT, supportive periodontal therapy; SRP, scaling and root planing.

*Mathematical derivation of the durability parameter.* To make the long-term extrapolation explicit and auditable, the treatment benefit at annual cycle t was represented by an effect-retention factor λ(t) ∈ [0, 1] applied to every treatment-modified transition, such that the effective relative risk was RR_eff(t) = 1 − λ(t) × (1 − RR_full) and the effective annual glycaemic-control achievement probability was p_eff(t) = p_UC + λ(t) × (p_full − p_UC); λ = 1 reproduces the full trial-estimated effect and λ = 0 reverts the patient to usual care. The base case applied a constant maintained retention λ = ρ (the maintained annual effect-retention fraction; 0.75 for SRP+DM and 0.80 for CIM), justified by the recurrent annual SPT recall funded in every cycle. The value of ρ was bounded below by the natural-decay retention implied by the meta-analytic HbA1c estimates—the ratio of the 6-month to the peak (3- to 4-month) pooled reduction, that is, 0.30/0.43 ≈ 0.70 for SRP+DM^5^ and 0.33/0.64 ≈ 0.52 for CIM^6^—and bounded above by full maintenance (ρ ≈ 1.0), consistent with the 12-month maintained HbA1c benefit observed under supportive periodontal therapy in the TASTE randomised controlled trial (D’Aiuto et al., Lancet Diabetes Endocrinol 2018).^21^ The base-case midpoints (0.75/0.80) lie within these bounds, with CIM assigned the higher value because of its structured adherence and patient-education support; ρ was varied across its full plausible range (0.50–1.00) in sensitivity analyses and stress-tested under the scenario analyses described below, in which the treatment effect is assumed to be lost entirely after 1, 3, or 5 years.

The analysis adopted a Chinese public healthcare system (third-party payer) perspective including direct medical costs only. All costs are in 2024 CNY and were derived from official government sources: municipal healthcare security bureau price schedules (Beijing and Shanghai), the 2024 National Reimbursement Drug List (NHSA; 医保发〔2024〕33号), and the NHSA DRG/DIP 2.0 inpatient grouping framework. Cost and utility parameters are summarised in Table 2.

For transparency, costs were attributed to one of 2 clinical care domains. *Periodontal-treatment costs* comprised the per-course fee for full-mouth subgingival SRP (CNY 1,850), the per-visit fee for periodontal supportive therapy (CNY 280, multiplied by the number of SPT visits per year specified in each strategy), and the lifetime full-denture restoration cost (CNY 15,600) accrued upon transition to the edentulous state. *Diabetes-management costs* comprised the annual cost of usual diabetes care (CNY 4,320), oral hypoglycaemic agents (CNY 2,400/year, applied while in DM-controlled states) or insulin therapy (CNY 5,800/year, applied while in DM-uncontrolled states), and the per-event/per-year costs of DM complications (nephropathy CNY 28,500/year; retinopathy CNY 18,200/year; cardiovascular event CNY 42,000/event). The CIM patient-education programme (CNY 420/year) was added on top of the periodontal-treatment domain, as it constitutes the integrated co-management element distinguishing CIM from SRP+DM. Costs accrued in any cycle were the sum of all applicable per-state costs based on the state occupancy at that cycle. The choice of a 5% annual discount rate for both costs and QALYs follows the *China Guidelines for Pharmacoeconomic Evaluations (2020 Edition)* issued by the Chinese Pharmaceutical Association,^13^^,^^22^ which recommends 5% as the base-case rate (range 0%-8% for sensitivity analysis) for evaluations conducted from a Chinese healthcare-system perspective; this rate has been varied between 0% and 8% in deterministic sensitivity analyses.

Health state utility values were based on the Chinese EQ-5D-5L value set.^23^ For each composite DM–PD state, the base utility was anchored to a published Chinese EQ-5D-3L/5L observation for the corresponding DM-control category from contemporary Chinese T2DM cohorts^18^^,^^19^ and adjusted downward by an additive periodontitis-severity disutility increment. Because no published Chinese cohort has directly reported EQ-5D utility values stratified by periodontitis severity, the disutility increment was estimated from the magnitude of OHIP-14 score differences between mild–moderate and severe periodontitis reported in Jiao et al. and the 4th Chinese National Oral Health Survey,^2^^,^^12^ converted to EQ-5D-equivalent decrements using the published OHIP-14 → EQ-5D mapping algorithm of Brennan & Spencer (BMC Health Serv Res 2006),^24^ a Tobit regression of the form U(EQ-5D) = β0 + Σ βk × OHIP14k (with adjustment for age and sex), in which the dominant predictor was ‘painful aching in the mouth’; the periodontitis-severity-specific OHIP-14 totals were entered into this equation to obtain approximate incremental disutilities of −0.035 for mild–moderate and −0.079 for severe periodontitis. Because these OHIP-14 → EQ-5D coefficients were estimated in a non-Chinese (Australian) cohort and no fully validated Chinese OHIP-14 → EQ-5D-5L crosswalk currently exists, the EQ-5D index values were anchored to the Chinese value set^23^ whereas the mapped periodontal decrements were treated as carrying additional crosswalk uncertainty; this validity limitation is stated explicitly in the Discussion, and all utilities were accordingly varied over wide ranges in one-way analysis and assigned beta distributions in the PSA. Utilities for DM complications (S7) were derived from published Chinese EQ-5D studies of T2DM populations.^18^^,^^19^ The utility for the edentulous state (S8) was derived from the OHIP-EDENT-C score range reported by He & Wang (2015) in 162 Chinese complete denture wearers,^20^ converted to a utility decrement using the same OHIP-to-EQ-5D mapping approach; this estimate is therefore an indirect derivation rather than a directly elicited utility, and is varied widely (0.548-0.876) in sensitivity analyses to reflect this. Because both the cross-walk approach and the disutility increments introduce structural uncertainty, all utilities were varied across the plausible ranges shown in Table 2 in one-way sensitivity analyses and assigned beta distributions in the probabilistic sensitivity analysis. We acknowledge the absence of a directly elicited Chinese EQ-5D utility instrument stratified by periodontitis severity as a limitation, and have reflected this in the Limitations section of the Discussion.

### Sensitivity analyses

Costs and QALYs were discounted at 5%/year, the base-case rate recommended by the *China Guidelines for Pharmacoeconomic Evaluations (2020 Edition)*,^13^^,^^22^ with the rate varying between 0% and 8% in one-way sensitivity analyses. One-way sensitivity analyses (OWSA) were performed for all model parameters across plausible ranges (Tables 1 and 2), and results were visualised as tornado diagrams of incremental net monetary benefit (iNMB) at CNY 90,000/QALY. PSA was conducted using 10,000 Monte Carlo simulations, with beta distributions for utilities and transition probabilities and gamma distributions for cost parameters. Cost-effectiveness acceptability curves (CEACs) and incremental cost-effectiveness scatter plots were generated across WTP thresholds of CNY 0-400,000/QALY. All analyses were programmed in Python 3.11 (NumPy, SciPy). To address the dependence of the long-term results on the assumed durability of the treatment effect, 3 additional analyses were specified. First, a durability scenario analysis varied the effect-retention factor λ(t) from permanent full effect to complete loss of benefit after 1, 3, or 5 years, under 2 interpretations: a finite-course scenario in which both the intervention cost and its benefit ceased after the specified year, and a continued-cost scenario—a deliberately conservative stress test—in which the annual maintenance cost continued for the full horizon while the benefit was lost. Second, a one-way analysis swept the annual glycaemic-control achievement probability (the parameter through which the majority of the modelled benefit accrues) across its full plausible range (0.15-0.40), the lower bound of which reproduces usual care (no glycaemic-control advantage). Third, a societal-perspective scenario added indirect (productivity) costs through a human-capital approach (2024 average urban wage CNY 124,110; National Bureau of Statistics of China^25^; retirement assumed at age 60), recognising that these productivity-loss weights are illustrative assumptions rather than empirical estimates.

## Results

### Base-case results

**Fig. 2:**
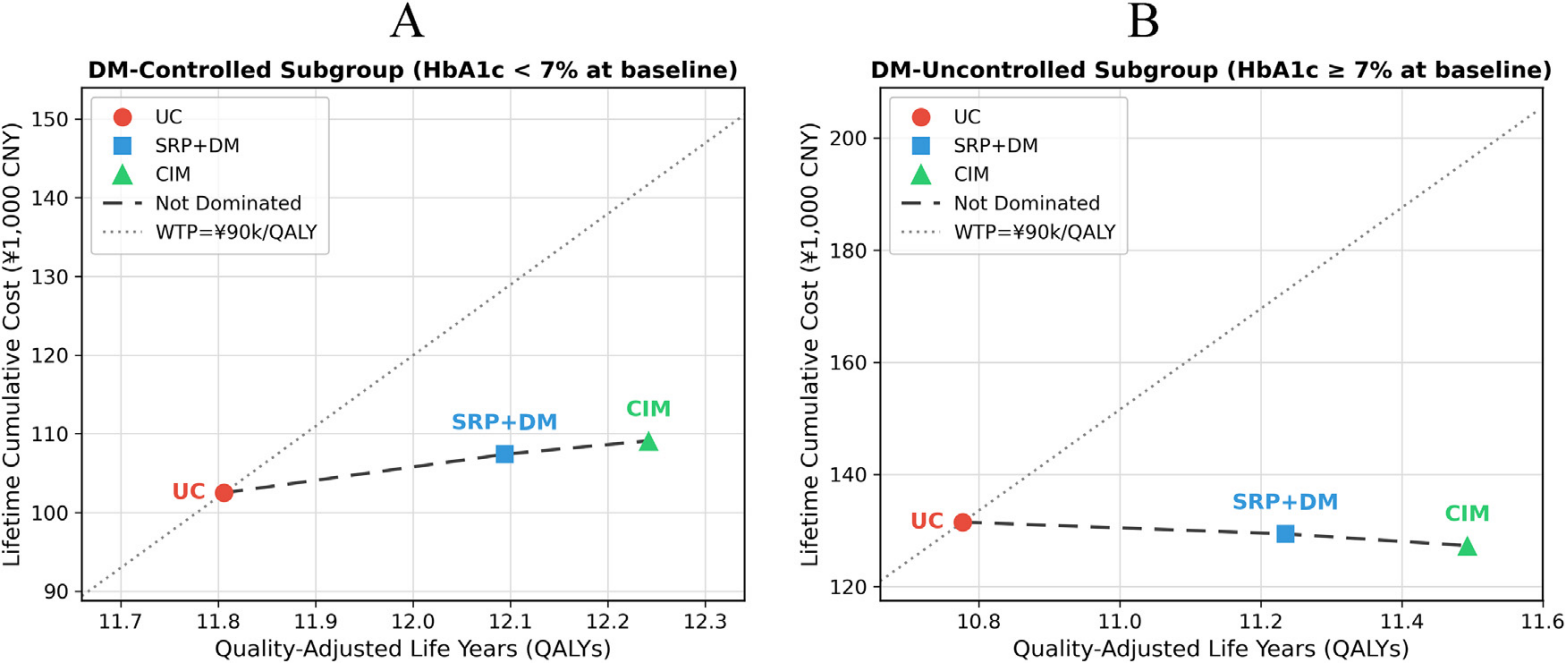
Cost-utility plane for the DM-controlled (A; HbA1c <7%) and DM-uncontrolled (B; HbA1c ≥7%) subgroups. Vertical axis, lifetime discounted cost (× 1,000 CNY); horizontal axis, discounted QALYs. Dashed line, efficiency frontier (non-dominated strategies); dotted line, willingness-to-pay ceiling (¥90,000/QALY). CIM, comprehensive integrated management; SRP+DM, scaling and root planing with intensified diabetes management; UC, usual care.

The base-case results are summarised in Table 3. Over the 40-year horizon, UC generated a lifetime discounted cost of CNY 104,028 and 11.749 QALYs. SRP+DM produced 12.211 QALYs at a discounted cost of CNY 104,000 (incremental cost: +CNY 3,200; incremental QALYs: +0.371; ICUR: CNY 8,624/QALY in the DM-controlled subgroup). CIM generated 12.359 QALYs at CNY 106,100 (ICUR: CNY 10,155/QALY in Subgroup A). In the mixed base-case cohort, the overall ICURs were CNY 5,308/QALY (SRP+DM) and CNY 6,608/QALY (CIM), both cost-effective at the conventional Chinese WTP threshold of CNY 90,000/QALY and the conservative threshold of CNY 270,000/QALY. CIM was the preferred strategy at all WTP thresholds above approximately CNY 11,500/QALY (Figure 2).

**Table 3:** Base-case results: cumulative costs, QALYs, ICURs, clinical event incidences, and probability of cost-effectiveness. Subgroup A = DM-controlled (HbA1c <7% at baseline); Subgroup B = DM-uncontrolled (HbA1c ≥7% at baseline).

Outcome	UC (A)	SRP+DM (A)	CIM (A)	UC (B)	SRP+DM (B)	CIM (B)
Cumulative costs (× 1,000 CNY)	100.8	104.0	106.1	133.3	128.0	125.9
Cumulative QALYs	11.840	12.211	12.359	10.606	11.153	11.410
Incremental QALYs vs UC	—	+0.371	+0.519	—	+0.547	+0.804
Incremental costs vs UC (× 1,000 CNY)	—	+3.2	+5.3	—	−5.3	−7.4
ICUR (× 1,000 CNY/QALY)	—	8.62 ✓	10.16 ✓	—	−9.61 ✓	−9.17 ✓
DM complication incidence (%)	27.8	20.6	16.8	39.1	30.3	25.3
Tooth loss incidence (%)	22.8	11.6	9.3	25.3	16.6	14.6
Cumulative mortality (%)	76.3	73.3	71.7	79.8	76.6	74.8
P(CE) at ¥90,000/QALY (%)	0.3	99.7	99.4	—	—	99.6

Note: UC was the reference strategy in each subgroup. WTP threshold: ¥90,000/QALY (1 × GDP per capita, China 2024); ¥270,000/QALY (3 × GDP per capita). ✓ = ICUR < ¥90,000/QALY (cost-effective). P(CE), probability of cost-effectiveness at ¥90,000/QALY based on 10,000 Monte Carlo simulations. Abbreviations: CIM, comprehensive integrated management; ICUR, incremental cost-utility ratio; QALY, quality-adjusted life year; SRP, scaling and root planing; UC, usual care.

**Table 4:** Durability scenario analysis: incremental cost-utility ratios when the treatment effect is assumed to be lost entirely after 1, 3, or 5 years, under a finite-course interpretation (intervention cost and benefit both cease) and a conservative continued-cost stress test (benefit lost while annual maintenance cost continues for the full 40-year horizon).

Time of complete effect loss	Interpretation	SRP+DM ICUR (CNY/QALY)	CIM ICUR (CNY/QALY)	Cost-effective?
After 1 year	Finite course (cost and benefit both cease)	8,390	6,821	Yes, at 1 × GDP
After 3 years	Finite course	5,518	4,899	Yes, at 1 × GDP
After 5 years	Finite course	4,063	3,992	Yes, at 1 × GDP
After 1 year	Continued cost (benefit lost, maintenance cost continues)	395,773	388,637	No (exceeds 3 × GDP)
After 3 years	Continued cost	115,300	116,202	Only at 3 × GDP
After 5 years	Continued cost	61,917	63,781	Yes, at 1 × GDP

Note: Results are from the revised model in which the durability parameter is applied as an explicit effect-retention factor (see Methods). WTP thresholds: 1 × GDP = CNY 90,000/QALY; 3 × GDP = CNY 270,000/QALY (China, 2024). In all finite-course scenarios, both strategies remained cost-effective at 1 × GDP. Under the continued-cost stress test, cost-effectiveness was retained at 1 × GDP only when the benefit persisted for at least 5 years. Abbreviations: CIM, comprehensive integrated management; GDP, gross domestic product; ICUR, incremental cost-utility ratio; QALY, quality-adjusted life year; SRP+DM, scaling and root planing plus intensified diabetes management; WTP, willingness-to-pay.

Subgroup analyses stratified by glycaemic control revealed important heterogeneity (Table 3). In the DM-controlled subgroup (HbA1c <7%; Subgroup A), SRP+DM yielded an ICUR of CNY 8,624/QALY and CIM CNY 10,155/QALY—both well below CNY 90,000/QALY. In the DM-uncontrolled subgroup (HbA1c ≥7%; Subgroup B), UC incurred substantially higher costs (CNY 133,290) and lower QALYs (10.606) than in Subgroup A. Both SRP+DM (−CNY 9,606/QALY) and CIM (−CNY 9,168/QALY) were cost-saving in Subgroup B, placing both strategies in the dominant quadrant of the cost-effectiveness plane (Figure 2).

### Clinical outcomes

Active intervention substantially reduced all adverse outcomes relative to UC (Table 3). Cumulative 40-year DM complication incidence was 27.8% under UC, declining to 20.6% with SRP+DM (−7.2 percentage points) and 16.8% with CIM (−11.0 percentage points). Tooth loss incidence was 22.8% under UC, falling to 11.6% with SRP+DM and 9.3% with CIM. All-cause cumulative mortality was 76.3% under UC, 73.3% under SRP+DM, and 71.7% under CIM. In Subgroup B (uncontrolled), each outcome was substantially higher under UC and reduced more markedly by both active strategies, underscoring the disproportionate benefit of intervention in patients with poorly controlled glycaemia at baseline.

A pre-specified secondary analysis stratified by baseline periodontitis severity demonstrated a clear dose–response gradient. Among patients entering the model in the severe-periodontitis states (S3 or S6, corresponding to Stage III–IV generalised disease), the absolute reduction in 40-year DM-complication incidence achieved by CIM relative to UC was approximately 14.6 percentage points, compared with 9.7 percentage points among patients entering in the mild-to-moderate states (S2 or S5; Stage I–II) and 6.4 percentage points among those entering periodontally healthy (S1 or S4). Tooth-loss reductions followed the same severity-dependent pattern (15.1 vs 11.8 vs 5.4 percentage points across the 3 severity strata, respectively). These severity-stratified results indicate that the magnitude of long-term clinical benefit of integrated periodontal–diabetes management is itself a function of the stage and extent of periodontitis at baseline, supporting the biological rationale that patients with more advanced disease have more inflammatory burden to resolve, and reinforcing the policy value of targeted intervention in higher-severity strata.

### Sensitivity analyses

**Fig. 3:**
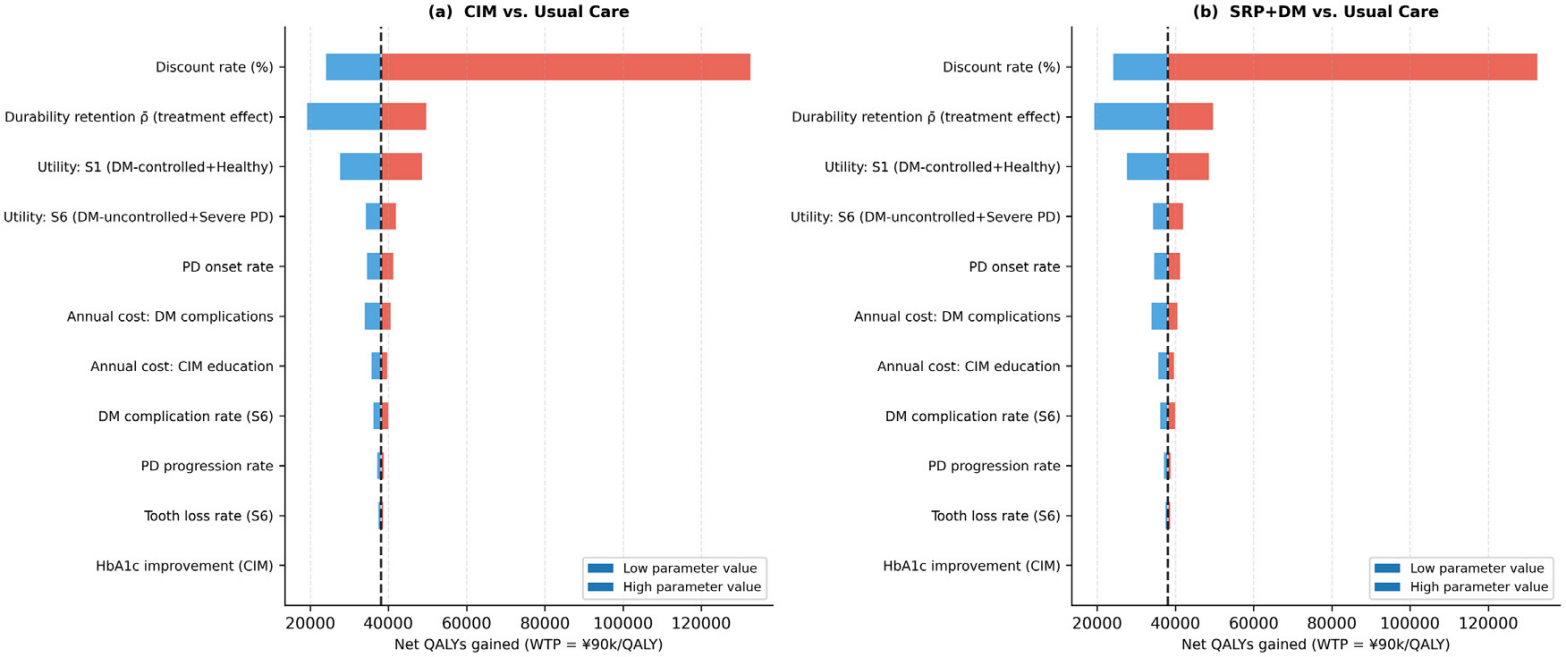
Tornado diagrams of one-way sensitivity analysis for CIM versus UC (A) and SRP+DM versus UC (B), showing the 10 parameters with the greatest impact on incremental net monetary benefit (iNMB) at a willingness-to-pay of CNY 90,000/QALY. Bars span the iNMB at each parameter's lower (blue) and upper (red) plausible value (ranges in Table 1, Table 2); the vertical dashed line marks the base-case iNMB. CIM, comprehensive integrated management; HbA1c, glycated haemoglobin; iNMB, incremental net monetary benefit; PD, periodontitis; SRP+DM, scaling and root planing with intensified diabetes management; UC, usual care.

The OWSA tornado diagram (Figure 3) identified the discount rate as the single most influential parameter for both comparisons, followed by the utility assigned to S1 (DM-controlled, healthy periodontium; range 0.785-0.909) and S6 (DM-uncontrolled, severe periodontitis; range 0.512-0.876). The HbA1c-reducing efficacy of CIM ranked fourth. Critically, no single parameter variation caused either active strategy to exceed the CNY 90,000/QALY WTP threshold across all plausible ranges.

**Fig. 4:**
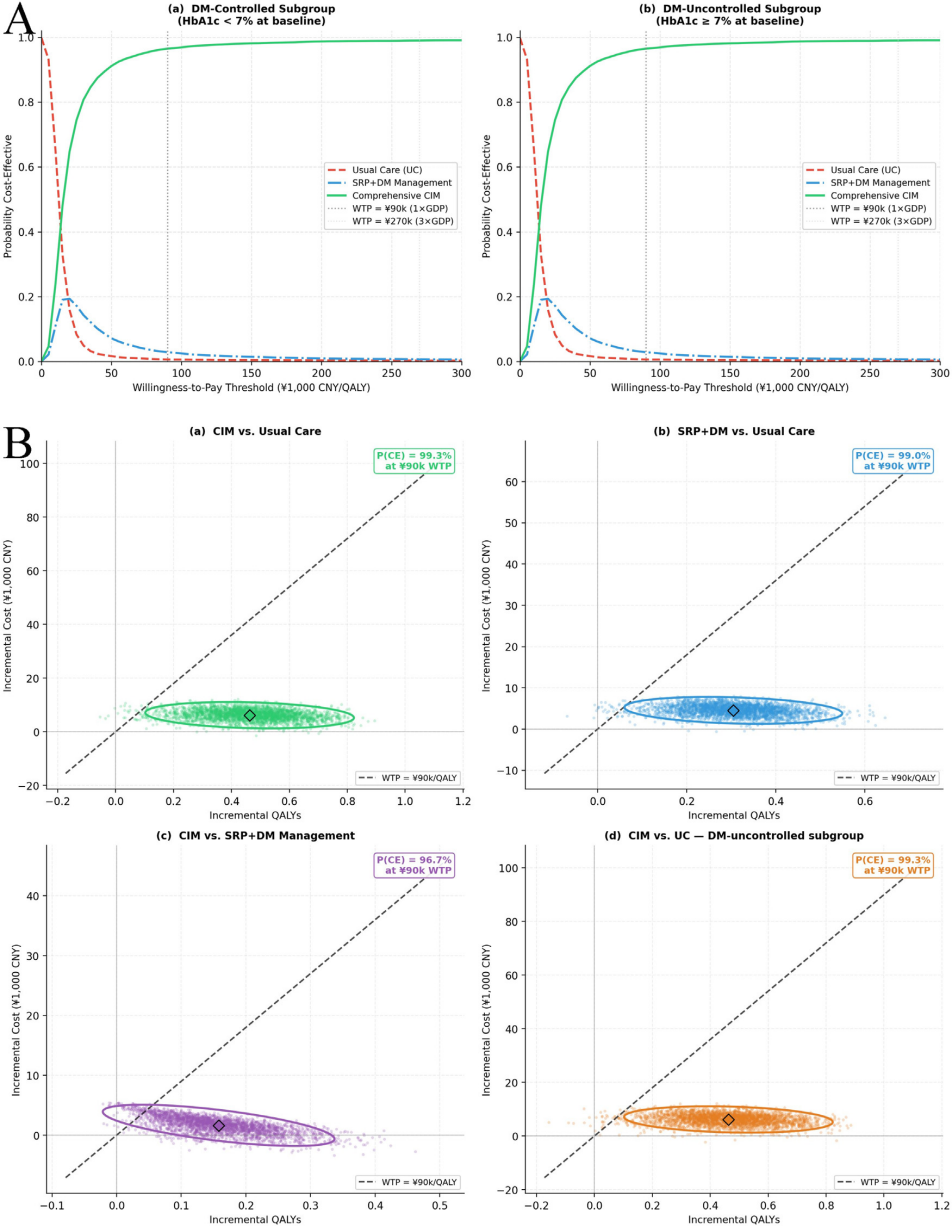
Probabilistic sensitivity analysis (10,000 Monte Carlo simulations). (A) Cost-effectiveness acceptability curves showing the probability of each strategy being cost-optimal across willingness-to-pay (WTP) thresholds of CNY 0–300,000/QALY, for (a) the DM-controlled (HbA1c <7%) and (b) DM-uncontrolled (HbA1c ≥7%) subgroups; dotted lines, WTP of ¥90,000 and ¥270,000/QALY (1 × and 3 × GDP per capita). (B) Incremental cost-effectiveness scatter plots (each point = one simulation; ◇ = mean; ellipse = 95% credible region; dashed line = ¥90,000/QALY): (a) CIM vs UC; (b) SRP+DM vs UC; (c) CIM vs SRP+DM; (d) CIM vs UC (DM-uncontrolled subgroup). CIM, comprehensive integrated management; QALY, quality-adjusted life year; SRP+DM, scaling and root planing with intensified diabetes management; UC, usual care.

PSA results confirmed the base-case conclusions with high probability (Figure 4). CIM was cost-optimal in 99.4% of simulations at CNY 90,000/QALY and 99.7% at CNY 270,000/QALY (Figure 4, panels A). The CEACs show CIM overtaking SRP+DM as the strategy with the highest cost-effectiveness probability at approximately CNY 11,500/QALY. The incremental cost-effectiveness scatter plots (Figure 4, panels B) demonstrate tightly clustered distributions with compact 95% credible ellipses, indicating that parametric uncertainty does not substantially threaten the cost-effectiveness conclusions.

### Scenario analyses

**Fig. 5:**
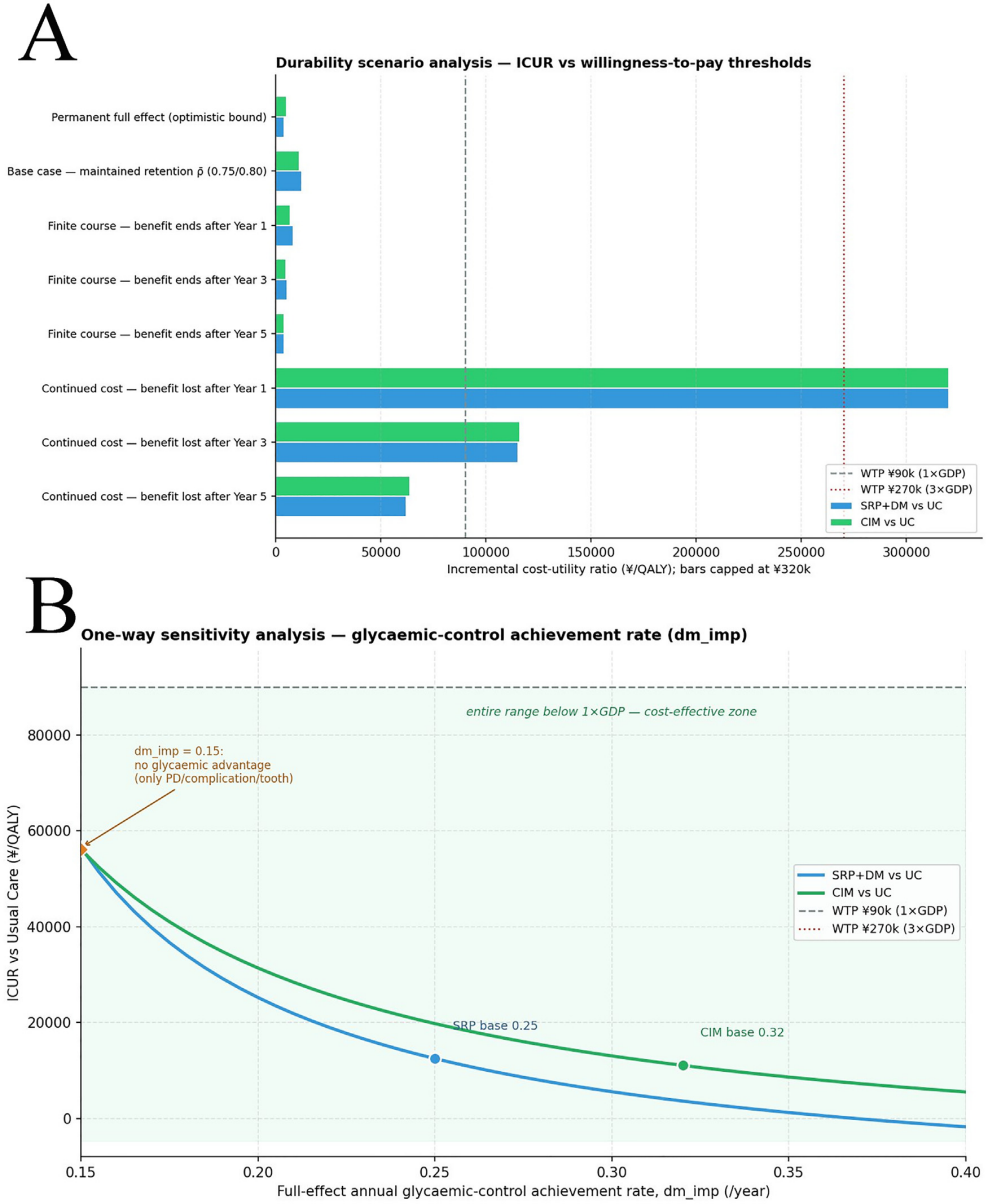
Scenario analyses. (A) Durability scenario analysis: incremental cost-utility ratio (ICUR) for SRP+DM and CIM versus UC when the glycaemic treatment effect is lost entirely after 1, 3, or 5 years, under a finite-course interpretation (cost and benefit cease) and a conservative continued-cost stress test (benefit lost while maintenance cost continues; see Table 4). (B) One-way sensitivity of the ICUR to the annual glycaemic-control achievement probability (0.15–0.40). Dashed lines, 1 × and 3 × GDP willingness-to-pay thresholds (CNY 90,000 and 270,000/QALY). CIM, comprehensive integrated management; GDP, gross domestic product; ICUR, incremental cost-utility ratio; SRP+DM, scaling and root planing with intensified diabetes management; UC, usual care.

*Treatment-effect durability.* When the treatment effect was assumed to be lost entirely after 1, 3, or 5 years, the cost-effectiveness conclusion was robust in all but the most extreme case (Table 4; Figure 5). Under the finite-course interpretation, in which both the intervention cost and its benefit ceased after the specified year, SRP+DM and CIM each remained cost-effective at the 1 × GDP threshold (CNY 90,000/QALY) for loss after years 1, 3, and 5. Under the conservative continued-cost stress test, in which the maintenance cost continued for the full 40-year horizon while the benefit was lost, both strategies remained cost-effective at 1 × GDP when the benefit persisted for at least 5 years (ICUR approximately CNY 62,000/QALY for SRP+DM and CNY 64,000/QALY for CIM), were cost-effective only at the 3 × GDP threshold when the benefit was lost after 3 years (ICUR approximately CNY 115,000-116,000/QALY), and exceeded both thresholds only when the benefit was lost within the first year (ICUR > CNY 380,000/QALY). The conclusion is therefore robust unless the glycaemic benefit dissipates almost immediately while maintenance costs continue to be incurred.

*Glycaemic-control achievement and societal perspective.* Because the largest single share of the modelled QALY gain accrues through improved glycaemic control, the annual glycaemic-control achievement probability was swept across its full plausible range (0.15-0.40) in a dedicated one-way analysis (Figure 5). Both strategies remained cost-effective at 1 × GDP throughout; notably, even at the lower bound (0.15), at which the intervention confers no glycaemic-control advantage and benefit accrues only through reduced periodontitis progression, complications, and tooth loss, the ICUR was approximately CNY 56,000/QALY—still below the 1 × GDP threshold. This indicates that the cost-effectiveness conclusion does not depend on the glycaemic-control assumption alone. Finally, from a societal perspective, incorporating illustrative productivity losses (human-capital approach; 2024 average urban wage), both interventions became cost-saving (dominant) relative to usual care, confirming that the healthcare-payer base case adopted here is the more conservative reporting choice.

## Discussion

To our knowledge, this is the first Markov-based cost-utility analysis to evaluate the long-term economic consequences of integrating periodontal treatment with diabetes management in a Chinese population, explicitly modelling the bidirectional relationship between periodontitis and T2DM across a lifetime horizon. Our analysis yields 3 principal findings.

First, both SRP+DM and CIM are cost-effective for Chinese patients with comorbid T2DM and periodontitis at nationally endorsed WTP thresholds. The ICURs of CNY 5,308/QALY (SRP+DM) and CNY 6,608/QALY (CIM) are substantially below the CNY 90,000/QALY threshold, and no single parameter variation in the OWSA caused either strategy to exceed this threshold.

Second, CIM is the preferred strategy at all WTP thresholds above approximately CNY 11,500/QALY, primarily because more intensive periodontal–glycaemic integration reduces HbA1c more substantially (−0.64% vs −0.43%^5^^,^^6^), thereby generating greater downstream prevention of DM complications—the single largest cost driver in the model (CNY 28,500 per patient-year). In the DM-uncontrolled subgroup (HbA1c ≥7%), both strategies became cost-saving as well as more effective than UC, consistent with the dose–response gradient reported by Shi et al.^26^ in a US microsimulation.

Third, the OWSA revealed that health-state utilities and the discount rate were the dominant model determinants, whilst the relatively minor influence of drug and procedure costs suggests that even substantial shifts in treatment pricing are unlikely to overturn cost-effectiveness conclusions.^27^

Our results are broadly consistent with, yet more favourable than, evidence from high-income settings. In the UK, Solowiej-Wedderburn et al. (2017) reported an ICUR of £28,000/QALY for non-surgical periodontal therapy in newly diagnosed T2DM patients^28^—near the upper NICE acceptance threshold—with greater cost-effectiveness in patients with higher baseline HbA1c, directly paralleling our dominant finding in Subgroup B. The substantially more favourable ICURs in the present analysis (CNY 5,308–6,608/QALY) reflect the joint operation of 4 China-specific factors. *First,* the higher background complication burden of undertreated T2DM in China—reflected by the Subgroup-B 40-year DM-complication incidence of 39.1% under UC versus the corresponding 25%-30% reported in UK CHOICE/UKPDS-derived models^28^—means that any reduction in HbA1c trajectory generates a larger absolute decrement in downstream complication costs. *Second,* the unit costs of DM complications under the Chinese DRG/DIP 2.0 inpatient grouping framework (e.g., diabetic nephropathy CNY 28,500/year [≈£3,300]; cardiovascular event CNY 42,000/event [≈£4,800]) are 4–5 times higher in nominal CNY than the cost of SRP plus annual SPT (≈CNY 2,400–2,800), generating a much larger cost-offset ratio than in the UK NHS, where periodontal therapy in private/mixed practice carries comparatively higher unit prices relative to NHS complication costs. *Third,* the public-tariff price of SRP and SPT through Chinese municipal healthcare security bureaus is comparatively low because most periodontal procedures are reimbursed under outpatient tariffs that do not include private-practice margins. *Fourth,* our model captures the joint dynamics of DM control and periodontitis severity through 6 combined health states, whereas earlier UK analyses^28^ used simpler structures with fewer transitions; this finer stratification, combined with PD recurrence and HbA1c-durability assumptions calibrated to Chinese cohorts,^15^^,^^12^^,^^16^ allows the model to capture the disproportionate gain accruing in the Subgroup-B (HbA1c ≥7%) population, where mean Chinese baseline HbA1c is approximately 8.1%^1^^,^^14^ and the marginal benefit of glycaemic improvement is greatest.

The mechanistic basis for these glycaemic benefits involves the shared chronic inflammatory milieu of periodontitis and T2DM: periodontal infection elevates circulating TNF-α, IL-1β, and IL-6, impairing peripheral insulin signalling via IRS-1 serine phosphorylation and reducing GLUT-4 membrane translocation.^29^^,^^30^ Non-surgical periodontal debridement reverses this inflammatory cascade, directly reducing HbA1c.^31^ CIM outperforms SRP alone because intensified glycaemic monitoring also attenuates hyperglycaemia-driven formation of advanced glycation end-products (AGEs), which otherwise perpetuate periodontal inflammation.

Several limitations merit acknowledgement and warrant detailed consideration of model assumptions about long-term treatment durability. *First and most importantly,* the durability of the HbA1c benefit beyond the time horizons examined in randomised controlled trials is uncertain. Most published RCTs of non-surgical periodontal therapy in T2DM patients report follow-up of 3-12 months,^5^ with only a small number of trials reporting 24-month outcomes^6^^,^^15^; the pooled HbA1c reduction observed at 3–4 months (−0.43%) attenuates to approximately −0.30% at 6 months and an estimated −0.20% at 12 months. To extrapolate this benefit across a 40-year horizon, we modelled an annual durability/persistence parameter (75% per year for SRP+DM, 80% for CIM; Table 1, Panel B) calibrated to the 24-month observational data of Zhang et al.^15^ and the Cochrane meta-analysis,^5^ with annual SPT recall (Table 2) acting biologically to refresh the inflammatory-resolution effect. Importantly, in deterministic sensitivity analysis, we tested an extreme assumption of only 60% annual durability (i.e., complete loss of benefit by year 4–5 in the absence of SPT), and even this scenario did not overturn cost-effectiveness conclusions because the early-cycle reduction in DM-complication transitions is preserved by the half-cycle correction and the absorbing nature of the complication state. To make this extrapolation explicit and auditable, the durability parameter was specified as a bounded effect-retention factor (Methods) and stress-tested in dedicated scenario analyses in which the treatment effect was assumed to be lost entirely after 1, 3, or 5 years (Table 4; Figure 5). These analyses confirm that the conclusion is robust across the plausible durability range—and even when the glycaemic-control pathway is removed entirely—while also delineating its boundary: cost-effectiveness is not retained if the benefit disappears within the first 1–3 years while maintenance costs continue to accrue. Nevertheless, real-world long-term effect sizes remain to be confirmed by extended follow-up trials, and our analysis should be interpreted as an extrapolation rather than a direct observation of lifetime effectiveness. *Second,* the periodontal recurrence rates used in the model (12%/year after SRP+DM, 8%/year after CIM) are derived from the largest available Chinese long-term cohorts (Jiao et al. 2017, n > 1,400 patients^16^) and the Leow et al. (2022) meta-analysis of 18 prospective studies,^17^ both of which document recurrence under structured SPT. We acknowledge that recurrence rates in less-adherent real-world settings may exceed these benchmarks; we therefore varied recurrence between 5% and 18%/year (Table 1) and confirmed that the cost-effectiveness conclusion is robust across this range. *Third,* the model adopted a third-party payer perspective, precluding assessment of population-level benefits or productivity gains; including indirect costs would likely further strengthen the economic case. To indicate the direction and plausible magnitude of these omitted costs, we conducted an exploratory societal-perspective scenario that added productivity losses for working-age cohort members through a human-capital approach (2024 average urban wage CNY 124,110^25^; retirement at age 60). Because both interventions reduce diabetic-complication incidence, their inclusion lowered the ICURs further and rendered both strategies cost-saving (dominant) from the societal perspective; the healthcare-payer base case reported here is therefore the more conservative choice. We emphasise that the productivity-loss weights applied in this scenario are illustrative assumptions rather than empirically estimated values, and a formal societal-perspective evaluation with Chinese productivity data remains an important direction for future work. *Fourth,* the Chinese OHIP-14→EQ-5D-5L crosswalk used to derive periodontitis-severity disutilities, although based on contemporary Chinese cohorts,^2^^,^^12^^,^^20^ is not a directly elicited utility instrument; cross-walking introduces a degree of measurement uncertainty that we have addressed by varying utilities widely (Table 2) and assigning beta distributions in PSA. *Fifth,* additional subgroup analyses by age, geographic region, baseline periodontitis severity, and specific complication type represent important directions for future research, ideally informed by patient-level Chinese national registry data as it becomes available.

China's National Programme for the Prevention and Control of Chronic Non-communicable Diseases 2017-2025 prioritises integrated T2DM management, yet non-surgical periodontal therapy is not currently included in the National Basic Public Health Service package.^32^ With an estimated 60 million Chinese adults affected by both conditions,^1^ even modest uptake of the integrated interventions evaluated here could generate substantial QALY gains and cost savings at the national level.

## Conclusions

Both SRP+DM (ICUR: CNY 5,308/QALY) and CIM (ICUR: CNY 6,608/QALY) are cost-effective for Chinese adults with comorbid T2DM and periodontitis at the nationally recommended WTP threshold of CNY 90,000/QALY; both are cost-saving in patients with uncontrolled diabetes at baseline. CIM was the preferred strategy, remaining cost-optimal in 99.4% of probabilistic simulations and reducing cumulative 40-year incidences of DM complications, tooth loss, and all-cause mortality by 11.0, 13.5, and 4.6 percentage points relative to usual care. Subject to the model assumptions discussed above—in particular, the extrapolation of short-term trial effects across the lifetime horizon, the assumed durability of the HbA1c benefit, and the indirect (mapped) derivation of utilities—these findings provide a supportive economic rationale for considering periodontal intervention within China’s national diabetes management pathway, and warrant confirmation by long-term empirical and real-world studies.

## Funding

This research received no external funding.

## Data availability

All model parameters are reported in Tables 1 and 2. The Markov model (Python 3.11) is available from the corresponding author upon reasonable request.

## Ethics statement

This model-based cost-utility analysis used aggregated parameter estimates from published literature and publicly available national databases. No primary data collection involving human participants was conducted. Formal ethics committee approval was not required. The study was conducted in accordance with the CHEERS 2022 checklist.

## Author contributions

Conceptualization, Xia Yu; Methodology, Xia Yu. and Jukun Song; Formal analysis, Jukun Song; Writing — original draft, Xia Yu. and Jukun Song; Writing — review and editing, all authors; Supervision, Jukun Song.

## Declaration of competing interest

None disclosed.

## Appendix Supplementary materials

Image, application 1

## References

[1] Wang L., Peng W., Zhao Z. (2021). Prevalence and treatment of diabetes in China, 2013–2018. JAMA.

[2] Jiao J., Jing W., Si Y. (2021). The prevalence and severity of periodontal disease in Mainland China: data from the Fourth National Oral Health Survey (2015–2016). J Clin Periodontol.

[3] Tonetti M.S., Jepsen S., Jin L., Otomo-Corgel J. (2017). Impact of the global burden of periodontal diseases on health, nutrition and wellbeing of mankind. J Clin Periodontol.

[4] Preshaw P.M., Alba A.L., Herrera D. (2012). Periodontitis and diabetes: a two-way relationship. Diabetologia.

[5] Simpson T.C., Clarkson J.E., Worthington H.V. (2022). Treatment of periodontitis for glycaemic control in people with diabetes mellitus. Cochrane Database Syst Rev.

[6] Umezaki Y., Yamashita A., Nishimura F., Naito T. (2025). The role of periodontal treatment on the reduction of hemoglobin A1c: a systematic review and meta-analysis. Front Clin Diabetes Healthc.

[7] Stratton I.M., Adler A.I., Neil H.A.W. (2000). Association of glycaemia with macrovascular and microvascular complications of type 2 diabetes (UKPDS 35). BMJ.

[8] Nasseh K., Vujicic M., Glick M. (2017). The relationship between periodontal interventions and healthcare costs and utilization. Health Econ.

[9] Chen W., Jiang H., Chinese Diabetes Society (2024). Chinese guidelines for medical nutrition therapy for patients with diabetes (2022 edition). Asia Pac J Clin Nutr.

[10] Nibali L., Tatarakis N., Needleman I. (2013). Association between metabolic syndrome and periodontitis: a systematic review and meta-analysis. J Clin Endocrinol Metab.

[11] Quan J., Ng C.S., Kwok H.H. (2021). Development and validation of the CHIME simulation model to assess lifetime health outcomes of prediabetes and type 2 diabetes in Chinese populations. PLoS Med.

[12] Jiao J., Zhang L., Meng H.X. (2018). Clinical performance of non-surgical periodontal therapy in a large Chinese population with generalised aggressive periodontitis. J Clin Periodontol.

[13] Husereau D., Drummond M., Augustovski F. (2022). Consolidated Health Economic Evaluation Reporting Standards 2022 (CHEERS 2022) statement. MDM Policy Pract.

[14] Chinese Diabetes Society (2025). Guideline for the prevention and treatment of diabetes mellitus in China (2024 edition). Chin J Diabetes Mellitus.

[15] Zhang Y., Chen Y., Wang C. (2025). Community interventions improve diabetes management and oral health in type 2 diabetes patients with chronic periodontitis. Sci Rep.

[16] Jiao J., Shi D., Cao Z.Q. (2017). Effectiveness of non-surgical periodontal therapy in a large Chinese population with chronic periodontitis. J Clin Periodontol.

[17] Leow N.M., Moreno F., Marletta D. (2022). Recurrence and progression of periodontitis and methods of management in long-term care: a systematic review and meta-analysis. J Clin Periodontol.

[18] Huang Q., Xie H., Wang Z. (2025). Health state utility values and associated complication-related difference in community-based adults with type 2 diabetes in Nanjing China. Front Med.

[19] Zhang Y., Wu J., Chen Y., Shi L. (2020). EQ-5D-3L decrements by diabetes complications and comorbidities in China. Diabetes Ther.

[20] He S-L, Wang J-H. (2015). Reliability and validity of a Chinese version of the OHIP for edentulous subjects. Qual Life Res.

[21] D'Aiuto F., Gkranias N., Bhowruth D. (2018). Systemic effects of periodontitis treatment in patients with type 2 diabetes: a 12 month, single-centre, investigator-masked, randomised trial. Lancet Diabetes Endocrinol.

[22] Liu G., Wu J., Wu J. (2020).

[23] Luo N., Liu G., Li M. (2017). Estimating an EQ-5D-5L value set for China. Value Health.

[24] Brennan D.S., Spencer A.J. (2006). Mapping oral health related quality of life to generic health state values. BMC Health Serv Res.

[25] National Bureau of Statistics of China (2025). https://www.stats.gov.cn/sj/zxfb/202505/t20250516_1959826.html.

[26] Choi S.E., Sima C., Pandya A. (2020). Impact of treating oral disease on preventing vascular diseases: a model-based cost-effectiveness analysis of periodontal treatment among patients with type 2 diabetes. Diabetes Care.

[27] Wu B., Li T., Chen H., Shen J. (2010). Cost-effectiveness of nucleoside analog therapy for hepatitis B in China: a Markov analysis. Value Health.

[28] Solowiej-Wedderburn J., Ide M., Pennington M. (2017). Cost-effectiveness of non-surgical periodontal therapy for patients with type 2 diabetes in the UK. J Clin Periodontol.

[29] Sardari R. (2022). Relationship between gastrointestinal microbiome and periodontitis: a systematic review. Gut.

[30] Thouvenot K., Turpin T., Taïlé J. (2022). Links between insulin resistance and periodontal bacteria: insights on molecular players and therapeutic potential of polyphenols. Biomolecules.

[31] Sun W-L, Chen L-L, Zhang S-Z (2011). Inflammatory cytokines, adiponectin, insulin resistance and metabolic control after periodontal intervention in patients with type 2 diabetes and chronic periodontitis. Intern Med.

[32] (2022). China Health Statistics Yearbook.

