# Supplementary material for: Cost-Effectiveness of Periodontal Intervention Combined with Diabetes Management in China: A Markov Analysis
Source: Int Dent J. 2026 Jul 18;76(5):109750. doi: 10.1016/j.identj.2026.109750 (PMC13393532; doi:10.1016/j.identj.2026.109750)
Supplement: Supplementary file 1 [file mmc1.docx]

**Supplementary Material**

*Cost-Effectiveness of Periodontal Intervention Combined with Diabetes Management in China: A Markov Analysis*

# **Parameter Source Detail**

This Supplementary Material provides a complete, transparent record of every parameter used in the Markov model, identifying (i) the underlying source literature, (ii) whether the value was directly extracted from a published source or derived/transformed for use in the model, and (iii) the rationale for any transformation. Parameters marked with a star (★) are model assumptions whose source literature did not report the parameter in the form required for the model and which were derived through the transformations described in the right-hand column. All such parameters were varied across wide plausible ranges in deterministic and probabilistic sensitivity analyses (Figure 3 and Figure 4 in the main manuscript).

## **Table S1. Transition probabilities and treatment-effect parameters**

| **Parameter (and Table 1 row)** | **Base value (range)** | **Source literature** | **Derivation rationale and transformation** |
| --- | --- | --- | --- |
| PD onset, S1→S2 (DM-controlled, healthy → mild–moderate PD) | 4.5%/yr (3.0–6.5) | Jiao et al. J Clin Periodontol 2021 [2] | Directly derived from the 4th National Oral Health Survey: prevalence of mild–moderate periodontitis (Stage I–II) in adults aged 35–44 vs 55–64 yields an approximate annualised onset hazard of 4.5%/yr; range reflects 95% CI of the underlying prevalence point estimate. |
| DM deterioration, S1→S4 (controlled → uncontrolled, healthy PD) | 8.0%/yr (5.5–11.0) | Wang et al. JAMA 2021 [1]; CDS Guideline 2024 [15] | Annual rate of loss of glycaemic control among Chinese T2DM patients on standard oral therapy, derived from Wang et al.’s longitudinal HbA1c-control rates between 2013–2018 (53.3% → 49.4%) and CDS-Guideline-reported deterioration patterns. |
| PD progression, S2→S3 (mild–moderate → severe PD) | 8.0%/yr (5.5–11.0) | Jiao et al. 2021 [2]; Simpson et al. Cochrane 2022 [5] | Progression rate from Stage I–II to Stage III–IV periodontitis under no/usual care. Calculated from age-stratified prevalence ratios in the 4th National Oral Health Survey. |
| Tooth loss, S2→S8 | 1.2%/yr (0.6–2.0) | Jiao et al. 2021 [2] | Annual tooth-loss incidence in mild–moderate Chinese periodontitis cohorts; range reflects within-cohort variability. |
| DM complication, S5→S7 (DM-uncontrolled, mild–mod PD → DM complications) | 3.5%/yr (2.0–5.5) | Quan et al. PLoS Med 2021 (CHIME) [11]; Xu et al. Lancet Public Health 2024 | Pooled annual incidence of any major DM complication (nephropathy, retinopathy, or cardiovascular event) in poorly-controlled Chinese T2DM, derived from the CHIME risk-equation set evaluated at HbA1c ≥7%. |
| DM complication, S6→S7 (DM-uncontrolled, severe PD → DM complications) | 6.8%/yr (4.5–9.5) | Quan et al. 2021 [11]; Xu et al. 2024 | Same source as S5→S7, with severity multiplier (~1.94×) reflecting added inflammatory burden of severe periodontitis on systemic inflammation in T2DM. |
| Death, S7→S9 (post-DM-complication mortality) | 5.5%/yr (3.5–8.0) | NHC China Health Statistics Yearbook 2022; WHO Life Tables 2021 | Age-standardised annual mortality among Chinese adults with active DM complications. Background mortality from age-specific life tables added additively. |
| Death, S8→S9 (post-tooth-loss mortality) | 3.0%/yr (1.8–4.5) | NHC Yearbook 2022; WHO Life Tables 2021 | Background mortality conditioned on edentulous state, with small upward adjustment for nutritional and frailty correlates of edentulism. |
| HbA1c reduction, SRP+DM | −0.43% (−0.59 to −0.28) | Simpson et al. Cochrane 2022 [5] | Pooled meta-analytic point estimate at 3–4 months from 30 RCTs (n=2,443). Confidence interval taken directly from published random-effects model. Sensitivity analysis also tested 6-month (−0.30%) and 12-month (−0.50%) values. |
| HbA1c reduction, CIM | −0.64% (−0.96 to −0.32) | Umezaki et al. Front Clin Diabetes Healthc 2025 [6] | Pooled point estimate at 3 months from 11 RCTs of integrated periodontal-plus-glycaemic-monitoring programmes; 95% CI taken directly from published random-effects model (I²=73%). |
| ★ Durability of glycaemic improvement, SRP+DM  [MODEL ASSUMPTION] | 75%/yr (60–90) | Calibrated to Zhang et al. Sci Rep 2025 [12]; Simpson et al. [5]; Umezaki et al. [6] | Source RCTs report HbA1c reductions only at 3, 6, and 12 months (e.g., Simpson −0.43%/3-4m, −0.30%/6m, −0.50%/12m). No source RCT directly measures year-on-year persistence beyond 12 months. We assumed an exponential-decay persistence rate of 75%/yr (i.e., 25% annual loss of effect in absence of SPT) based on the 6-month-to-baseline ratio in Zhang et al. 2025 (T2 vs T0). The plausible range 60–90% spans complete loss of benefit by year 4 (lower bound) to near-permanent maintenance with effective SPT recall (upper bound). |
| ★ Durability of glycaemic improvement, CIM  [MODEL ASSUMPTION] | 80%/yr (65–90) | Calibrated to Zhang et al. 2025 [12]; Umezaki et al. [6] | Same exponential-decay rationale; CIM assumed slightly higher annual persistence (80% vs 75%) because the integrated dietary/educational components reinforce behavioural change. Sensitivity range overlaps with SRP+DM range to test robustness if the difference is overstated. |
| ★ PD recurrence after SRP, SRP+DM  [MODEL ASSUMPTION] | 12.0%/yr (8.0–18.0) | Annualised from cumulative data in Leow et al. J Clin Periodontol 2022 [17]; Jiao et al. 2017 [16] | Leow et al. 2022 reports ONLY 5–20-year cumulative prevalence of CAL loss ≥2 mm (24.8%, 95% CI 11–38%) and tooth loss (9.6%, 95% CI 5–14%) under structured supportive periodontal care; no annual rate is published. We applied a constant-rate exponential transformation across the 12.5-year midpoint, yielding ≈10–12%/yr CAL-recurrence equivalent. Adjusted upward to 12%/yr for SRP+DM to reflect lower SPT-adherence in routine community care vs the largely university/specialist-care cohorts in Leow et al. Sensitivity range 8–18%/yr. |
| ★ PD recurrence, CIM  [MODEL ASSUMPTION] | 8.0%/yr (5.0–13.0) | Same as above; calibrated lower for intensive co-management | Same derivation as SRP+DM, adjusted downward to 8%/yr to reflect the tighter SPT recall and patient-education schedule in the CIM arm. Sensitivity range overlaps with SRP+DM range; PSA-derived results were robust to ±50% variation. |

*DM = diabetes mellitus; PD = periodontitis; SRP = scaling and root planing; CIM = comprehensive integrated management; SPT = supportive periodontal therapy; CAL = clinical attachment loss; DRG = diagnosis-related group; CHIME = Chinese Hong Kong Integrated Modelling and Evaluation. ★ marks model assumptions, not directly reported in source literature.*

## **Table S2. Cost parameters (2024 CNY)**

| **Parameter (and Table 2 row)** | **Base value (CNY/yr)** | **Source** | **Derivation rationale** |
| --- | --- | --- | --- |
| Usual Care (DM management) | 4,320 (3,200–5,800) | NHSA fee schedule 2024 (Beijing/Shanghai) | Directly extracted: weighted-average annual outpatient T2DM follow-up tariff (4 quarterly visits + routine HbA1c/glucose monitoring) under the urban employee basic medical insurance schedule. |
| Subgingival SRP, full mouth (per course) | 1,850 (1,400–2,500) | Public hospital fee schedule | Directly extracted: tariff for full-mouth subgingival SRP (CPT-equivalent: 4 quadrants of root planing) under the Beijing/Shanghai municipal stomatology fee schedule. |
| Periodontal maintenance (SPT, per visit) | 280 (200–380) | Outpatient fee schedule | Directly extracted: tariff for one SPT visit (re-examination + supragingival/subgingival professional biofilm removal). Annual SPT cost = 280 × number of visits per year (4 in Y1, 2 from Y2 onward). |
| CIM patient education programme | 420 (300–600) | Community health centre rate | Directly extracted: tariff for structured annual diabetes-periodontal co-management education session in community health centre. |
| Oral hypoglycaemic agents | 2,400 (1,800–3,200) | NHSA Reimbursement Drug List 2024 | Weighted-average annual cost across the most commonly prescribed agents (metformin, sulphonylureas, DPP-4 inhibitors); reimbursement tier-2 prices. |
| Insulin therapy | 5,800 (4,500–8,000) | NHSA Drug List 2024 | Weighted-average annual cost across basal-bolus and pre-mixed insulin regimens for DM-uncontrolled patients. |
| Diabetic nephropathy management | 28,500/yr (22,000–38,000) | NHSA DRG/DIP 2.0 2024; Liu et al. Front Public Health 2023 [18] | Directly extracted: average inpatient + outpatient annual cost in the DRG bundle (KK-31, diabetic nephropathy). Cross-validated against Liu et al.'s claims-data analysis (CNY 27,800–29,200/yr range). |
| Diabetic retinopathy treatment | 18,200/yr (14,000–25,000) | NHSA DRG/DIP 2.0 2024; Huang et al. PLoS One 2016 [19] | Directly extracted: average annual cost across non-proliferative + proliferative DR treatment, including anti-VEGF/laser/surgery. |
| Cardiovascular event (per event) | 42,000/event (32,000–56,000) | NHSA DRG/DIP 2.0 2024; Liu et al. 2023 [18] | Directly extracted: weighted-average DRG cost across MI/stroke/PCI events. |
| Full denture restoration (lifetime) | 15,600 (10,000–22,000) | Beijing/Shanghai MHSB prosthetics 2024 | Directly extracted: tariff for one course of removable complete dentures (upper + lower) including impressions, fittings, and follow-up adjustments. |

*All costs in 2024 Chinese Yuan (CNY). NHSA = National Healthcare Security Administration; MHSB = Municipal Healthcare Security Bureau; DRG = diagnosis-related group; DIP = diagnosis-intervention packet; SPT = supportive periodontal therapy; PCI = percutaneous coronary intervention.*

## **Table S3. Health-state utility values**

| **Health state** | **Base utility (range)** | **Source** | **Derivation rationale** |
| --- | --- | --- | --- |
| S1: DM-controlled + Healthy PD | 0.847 (0.785–0.909) | Luo et al. Value Health 2017 [20] | Directly extracted: Chinese EQ-5D-5L value-set anchor for DM-controlled population without periodontal disease (modal health profile 11111 / 11211, weighted by Chinese T2DM-controlled cohort prevalence). |
| S2: DM-controlled + Mild–mod PD | 0.812 (0.670–0.954) | Anchored to S1 [20]; PD-disutility from Jiao et al. 2021 [2], 2018 [13] | INDIRECT DERIVATION. S1 utility (0.847) minus an additive periodontitis disutility increment of −0.035, derived from the OHIP-14 score difference between healthy and Stage I–II periodontitis cohorts in Jiao et al. (mean OHIP-14 score 4.5 vs 8.7), converted to EQ-5D-equivalent decrement using a published OHIP-to-EQ-5D mapping coefficient (~0.011 utility per OHIP-point). Wide range reflects mapping uncertainty. |
| S3: DM-controlled + Severe PD | 0.768 (0.600–0.936) | Anchored to S1 [20]; PD-disutility from Jiao et al. [2, 13] | INDIRECT DERIVATION. S1 utility minus an additive disutility of −0.079 for Stage III–IV (severe) periodontitis, calculated by the same OHIP→EQ-5D mapping (mean OHIP-14 score 8.7 vs 15.9). |
| S4: DM-uncontrolled + Healthy PD | 0.798 (0.660–0.936) | Luo et al. 2017 [20]; Zhang et al. Diabetes Ther 2020 [22] | Directly extracted: weighted average of EQ-5D utility for poorly-controlled Chinese T2DM (Zhang 2020, n=9570 hospital cohort) anchored to Chinese EQ-5D-5L value-set. |
| S5: DM-uncontrolled + Mild–mod PD | 0.749 (0.591–0.907) | Anchored to S4 [20, 22]; same disutility approach | INDIRECT DERIVATION. S4 utility minus −0.049 disutility for mild–moderate PD (slightly larger than for DM-controlled state, reflecting interaction effect). |
| S6: DM-uncontrolled + Severe PD | 0.694 (0.512–0.876) | Huang et al. Front Med 2025 [21]; Zhang et al. 2020 [22] | INDIRECT DERIVATION. Anchored to Huang Q. et al. 2025 community-based Nanjing T2DM utility for those with multiple complications (n=2,153, mean utility ≈0.785) and adjusted further for severe PD via the OHIP→EQ-5D mapping. Wide CI reflects the compounded uncertainty of mapping plus interaction effect. |
| S7: DM complications | 0.631 (0.435–0.827) | Huang et al. 2025 [21]; Zhang et al. 2020 [22] | Directly extracted: weighted average of EQ-5D-3L utility decrements for nephropathy/retinopathy/CVD events from Zhang et al. 2020 (mean coefficient −0.20 vs no-complication state), anchored to Chinese EQ-5D-5L value-set baseline. |
| ★ S8: Tooth loss / Edentulous  [INDIRECT DERIVATION] | 0.712 (0.548–0.876) | Mapped from He & Wang Qual Life Res 2015 [23] | He & Wang 2015 is a psychometric validation study of the Chinese OHIP-EDENT-C in 162 Chinese complete-denture wearers (Cronbach α=0.972, ICC=0.763); it does NOT directly report EQ-5D utility values. We mapped the reported OHIP-EDENT-C summary score range (mean ~26 across the cohort) to an EQ-5D-equivalent decrement of approximately −0.13 from the Chinese age-matched general-population norm (~0.84 for adults aged ≥60), yielding 0.712. The wide sensitivity range (0.548–0.876) reflects this measurement uncertainty. We acknowledge in the Limitations section of the main manuscript that no Chinese cohort has directly elicited EQ-5D utilities for the edentulous state. |
| S9: Death | 0.000 (fixed) | Convention | Standard convention for absorbing death state. |

*All utilities anchored to the Chinese EQ-5D-5L value set (Luo et al. 2017). EQ-5D = EuroQol 5-dimension; OHIP-14 = Oral Health Impact Profile-14; OHIP-EDENT-C = Chinese version of OHIP for edentulous subjects; PD = periodontitis. ★ marks indirect derivations, where source literature did not directly report an EQ-5D utility value for the relevant health state and an OHIP-to-EQ-5D mapping was applied. All utilities varied across the listed ranges in deterministic and probabilistic sensitivity analyses (beta distributions in PSA).*

## **Notes on parameter classification**

Parameters in this manuscript fall into three categories:

(1) Directly extracted from published Chinese national or contemporary Chinese cohort/meta-analytic data — e.g., DM prevalence, periodontitis prevalence, CHIME-derived complication risk equations, NHSA fee tariffs, and pooled HbA1c reductions from Cochrane (Simpson 2022) and Frontiers (Umezaki 2025) meta-analyses. These are the majority of parameters and are presented in the right-hand column of Tables S1–S3 with the phrase 'Directly extracted'.

(2) Indirectly derived through a published mapping/transformation — e.g., the OHIP-to-EQ-5D mapping used to obtain periodontitis-severity disutility increments, and the cumulative-to-annual rate transformation used for periodontitis recurrence. These transformations introduce structural uncertainty that has been propagated through wide sensitivity-analysis ranges (typically ±30–50% of the point estimate) in both deterministic OWSA (Figure 3) and probabilistic PSA (Figure 4).

(3) Model assumptions calibrated to short-term trial data — specifically the annual durability/persistence parameters for HbA1c improvement, which are model assumptions because no source RCT directly measures year-on-year persistence beyond 12 months. These are calibrated to the trial-level 3-, 6-, and 12-month point estimates using a constant-rate exponential decay model and varied across wide plausible ranges (60–90% per year).

This stratified approach to parameter sourcing follows CHEERS 2022 guidance (Husereau et al. 2022) on transparent reporting of parameter inputs in health economic evaluations and is consistent with the China Guidelines for Pharmacoeconomic Evaluations (2020 Edition) recommendations on sensitivity analysis.
